# Editorial: COVID-19: School and community feeding programs for children and young people

**DOI:** 10.3389/fpubh.2022.1066198

**Published:** 2022-12-06

**Authors:** Margaret A. Defeyter, Lesley Drake, Donald A. P. Bundy

**Affiliations:** ^1^The Healthy Living Lab, Northumbria University, Newcastle upon Tyne, United Kingdom; ^2^Partnership for Child Development, Imperial College London, London, United Kingdom; ^3^Research Consortium for School Health and Nutrition, London School of Tropical Hygiene and Medicine, University of London, London, United Kingdom

**Keywords:** school health and reading program (SHRP), COVID-19, food insecurity, economic value, government investment

The papers included in this Research Topic clearly show how schools, and school feeding programs, are central to all educational recovery programs in building back better from the COVID-19 pandemic. The school closures in response to the COVID-19 pandemic caused a global crisis in the education sector and saw 370 million children worldwide lose access to their daily school meal. In parallel, an additional 100 million people were pushed below the $1.90 poverty threshold in 2020, with the increase in poverty concentrated in the Africa region (Verguet et al.). These concurrent events highlight the need to build-back education systems that can deliver age appropriate and country specific health services, including mental health and wellbeing services. Increasing rates of child poverty and malnutrition combined with increases in the cost of living across the globe represent a significant risk to children's physical and mental health, wellbeing, and learning and school feeding programs are gaining increasing recognition for their twin roles as a longer social protection investment as well as acting as a productive safety net for children and their families in the short term (1, 2).

Many school meal programs are associated with complementary school health programs (e.g., school worming and nutritional education). Importantly, such multi-faceted programs (3) are among the most effective large-scale interventions available to governments, wishing to transform educational and child health outcomes. Research has shown that well-designed and well-implemented school meal programmes, and holiday programs, have the potential to raise educational attainment, reduce household food insecurity, promote social equity, and reduce health inequalities (1, 2).

The COVID-19 pandemic has resulted in a renewed focus on children's and young people's health and wellbeing. The interaction between health and education is one of the driving forces for developing the human capital that drives shared economic prosperity, and emerging research suggests that investing in the learner in terms of health and education is vital. Aggregated at the national level, investments in human capital drive national economies, with over 70% of the wealth of high-income countries attributed to human capital compared to 40% in low-income countries. School age children and young people, ages 5–18/19 years, require specific attention. It is during the first 8,000 days of life that children and young people undergo physical, emotional, and cognitive changes and development (Schultz and Ruel-Bergeron). We suggest that the school system offers an exceptionally cost-effective platform though which to deliver an integrated package of health and nutrition services, such as school meals, iron and folic acid supplementation, vision screening, amongst others.

Evidence from low-income countries shows that school meals provide an incentive for families to ensure their families regularly attend school. Likewise, in middle-to-high income countries, research has shown that nutritious school breakfast clubs drive attendance and punctuality. The 2016 International Commission on Financing Global Education Opportunity identified school meals as a highly effective non-teaching practice to increase access and learning outcomes, and a recent UN agency report ranked school meals among interventions with the strongest evidence of impact on equity and inclusion in education. The benefits are felt most acutely by vulnerable students and girls. In low- and lower-middle-income countries, about 300 million schoolchildren have iron-deficiency anemia, causing them to lose some six IQ points per child. For these reasons nearly every country in the world provides some form of national school meal programme, with nearly half of primary schoolchildren in lower-middle income countries eating a meal at school.

School meals are cost-effective and cost-beneficial because of the returns from substantial benefits across multiple sectors. The single intervention of school feeding can have effects across at least four different sectors: agriculture, education, health and nutrition, and social protection, with $9 in returns for every $1 invested. School feeding programmes that procure food locally can offer additional benefits for smallholder farmers, supporting local food production and economies, and promoting sustainable local markets for diverse, nutritious foods (4, 5). School meals also serve as an important safety net, supporting families' efforts to counter the current threats to the food system and supply chain, something that is becoming increasingly evident across the world.

Most of the “business as usual” education interventions do not result in measurable improvements in education outcomes—e.g., over half the education interventions reviewed in a World Bank working paper (6) and by the Global Education Evidence Advisory Panel (7) show no effectiveness.

Of course, child development does not just take place whilst children and young people are in school. The recent focus on out-of-school programs, such as the UK's Holiday Activities and Food (HAF) program demonstrates the importance of providing essential nutrition and activities for children during the school holidays. Supporting families with well-designed programs that run through the year will help to ameliorating the negative effects of household food insecurity [(8); Shinwell and Defeyter]. Furthermore, holiday programs, such as HAF can quickly be adapted to provide children and young people with quality, nutritious meals during periods of school closure [(9); Bayes et al.].

This Research Topic provides examples from across the globe where school and school holiday feeding programs are making a difference to children's and young people's lives. The papers included in this Research Topic provide a clear summary regarding the number of children participating in school meals programs. Two papers specifically explore how to monitor school meal programs and a methodology to conduct an economic evaluation. Schultz and Ruel-Bergeron provide an overview of how monitoring indicators can be selected from across global school health and nutrition programmes. Importantly, this paper discusses: why monitor, what to measure, how to measure, and who measures. Verguet et al. provide an economic evaluation methodology to estimate the cost and benefits of feeding programmes across four sectors: health and nutrition; education; social protection; and the local agricultural economy. They applied this methodology to school health and nutrition programs from 14 countries and concluded that the overall benefits of school feeding programs are several times greater than public investment. Alongside exploring school food programs, Shinwell and Defeyter and Bayes et al. explore how the Holiday Activities and Food programme (HAF) adapted to provide meals during the COVID-19 pandemic, and how food insecurity appears to be a constant factor in the lives of low-income households within England and Northern Ireland. Both papers refer to the how organizations delivering holiday activities and food struggle to acquire funding to match the need of people in under-served communities within England and Northern Ireland. Finally, the article by Lemuel et al. presents evidence on the relationship between education level, relationship status and mental health during COVID-19, and reminds the reader of the importance of considering different demographic factors in public health interventions. Together these papers makes a strong health, educational and economic case for governments to invest in well-designed school meals and school holiday programs, ensuring that such programs are designed and implemented to invest in the physical and mental wellbeing of the learner.

## Author contributions

All authors listed have made a substantial, direct, and intellectual contribution to the work and approved it for publication.
